# Physiological responses to physical activity: A multi-sensor wearable study across activity intensity, age, gender, body weight, and BMI variability

**DOI:** 10.1097/MD.0000000000044725

**Published:** 2025-10-03

**Authors:** Eyad Talal Attar

**Affiliations:** a Department of Electrical and Computer Engineering, Faculty of Engineering, King Abdulaziz University, Jeddah, Saudi Arabia.

**Keywords:** blood pressure, cardiovascular monitoring, demographics, heart rate, machine learning, oxygen saturation, physical activity, wearable sensors

## Abstract

Designing exercise interventions and assessing cardiovascular health depend on an understanding of how physiological parameters react to physical activity. Twenty-two healthy adults (ages 20–53, both sexes, varying fitness levels) participated in this study to examine the effects of 3 activity levels on heart rate (HR), systolic blood pressure (SBP) and diastolic blood pressure, and arterial oxygen saturation (peripheral oxygen saturation). The activities levels were sitting (rest), walking (~3 metabolic equivalents of tasks), and running (~7 metabolic equivalents of tasks), and each lasted roughly 5 minutes. For ongoing physiological monitoring, participants wore a specially designed multi-sensor device that combined force, photoplethysmography, inertial measurement unit, and electrocardiogram sensors. The physiological signals utilized in this investigation were supplemented by internal sensor validation and sourced from open-source datasets made available through PhysioNet. Higher activity intensity was associated with significant increases in HR (*P* < .001) and SBP (*P* < .01), according to repeated measures analysis of variance. HR increased stepwise across all activity conditions, while SBP significantly rose from sitting to running. Peripheral oxygen saturation values showed a slight postexercise decline, among older participants (*P* = .03), but they stayed within normal ranges (>95%). Age and weight were found to have moderate relationships with cardiovascular responses, specifically SBP, according to regression analysis. The random forest model’s low predictive accuracy (*R*^2^ = 0.057) when predicting HR from physiological and demographic inputs emphasized the necessity for additional behavioral or contextual data. These results demonstrate the impact of age gender weight and body mass index on cardiovascular dynamics while showcasing how multi-sensor wearables detect individual physiological responses. This work’s implications support customized exercise recommendations along with hypertension risk assessment and endurance training approaches for both clinical and athletic populations.

## 1. Introduction

An understanding of physiological responses to physical activity forms the basis for treating chronic conditions like obesity and hypertension while managing cardiovascular health and developing targeted exercise interventions. The measurement of physiological indicators like heart rate (HR) blood pressure (BP) and peripheral oxygen saturation (SpO₂) helps assess how the respiratory and cardiovascular systems adapt to the dynamic load imposed by physical activity. The body’s ability to meet metabolic demands during various physical exertion levels is assessed through metrics influenced by exercise intensity age gender and body composition.^[1,2]^

Individuals widely accept HR as a reliable indicator of exercise intensity. The body’s need for oxygen causes cardiac output to rise which results in HR increasing linearly with physical workload. Exercise conditions trigger sympathetic-mediated vasoconstriction while stroke volume increases, causing systolic blood pressure (SBP) to rise.^[3]^ The phenomenon of peripheral vasodilation works to maintain diastolic blood pressure (DBP) at stable levels or cause slight reductions instead.^[4]^ These responses show individual variation of age-related alterations such as decreased arterial flexibility and diminished baroreceptor responsiveness cause older adults to exhibit heightened cardiovascular reactions during physical activity.^[5]^

SpO₂ serves as another vital measurement by representing the proportion of hemoglobin molecules carrying oxygen. The SpO₂ values maintain their normal range above 95% during mild-to-moderate physical activities. In situations of intense physical activity or among individuals with cardiopulmonary limitations, increased tissue oxygen extraction combined with potential lung diffusion limitations can lead to temporary SpO₂ decreases.^[6]^ The real-time observation of these modifications delivers critical data regarding recovery progress, potential danger conditions, and cardiopulmonary performance.

Wearable sensor technologies have enabled significant advancements in tracking physiological parameters during real-world conditions. The integration of electrocardiographic (ECG) leads with photoplethysmographic (PPG) sensors and inertial measurement units (IMUs) in devices enables continuous multi-modal data collection during physical activity.^[7,8]^ Through scalable noninvasive methodologies, these tools enable the assessment of physiological dynamics beyond clinical settings, which expands access to individualized health information.^[9]^

Despite these advances, many studies employing wearable systems are limited by their narrow participant demographics and artificial experimental environments. In particular, variability related to age, gender, weight, and body mass index (BMI) is often underrepresented, which limits the generalizability of findings. For instance, Cornelissen and Fagard studied BP responses to endurance training but focused on relatively homogeneous groups.^[10]^ Similarly, Giggins et al assessed motion during rehabilitation exercises using inertial sensors, but under controlled laboratory conditions.^[11]^ Wang et al reviewed multi-modal sensor integration but noted limited use in demographically diverse, free-living populations.^[12]^ Accelerometry was used by Godfrey et al to measure physical activity, but they did not establish a direct connection between movement data and cardiovascular markers.^[13]^ Comprehensive studies that incorporate synchronized physiological and biomechanical data from a diverse participant group engaging in various intensities of activity are still lacking.

By examining the physiological effects of 3 activity levels – sitting, walking, and running – on HR, BP, and SpO₂ in a demographically diverse sample of healthy adults, the current study fills this knowledge gap. A specially crafted wearable device gathered multi-sensor data, including ECG, PPG, IMU and force sensors in semi-controlled provide ecologically valid environments. Each activity spanned approximately 5 minutes, while intensity levels were estimated using metabolic equivalents, where sitting represented rest, walking approximated 3 metabolic equivalents of tasks (METs), and running reached about 7 METs. The study of physiological responses during activity transitions becomes feasible through the integration of variables such as age gender and body weight to assess both individual and combined effects.

This study hypothesizes that HR and SBP increase significantly with physical activity intensity. That age and weight positively correlate with both baseline and activity-induced changes in cardiovascular parameters. Also, SpO₂ may decline slightly after high-intensity activity, particularly among older individuals. These hypotheses are grounded in established physiological research and seek to enhance our understanding of how wearable technology can be used to evaluate real-time cardiovascular dynamics in healthy populations.

## 2. Methods

### 2.1. Participants

A group of 22 healthy adult volunteers, consisting of 12 males and 10 females recorded an average age of 34. 6 ± 8. 9 years, weights of 74. 2 ± 12. 4 kg, and BMIs of 24. 8 ± 3. 1 kg/m² participated in this study. The selected participants from the general population exhibited a wide range of body compositions and fitness levels. None of the people had any known metabolic, pulmonary, or cardiovascular conditions. The ability to perform all 3 activity tasks physically was a requirement for inclusion. A history of respiratory illness, heart disease, or hypertension was one of the exclusion criteria.

To find participants, convenience sampling was employed. For repeated measures analysis of variance (ANOVA), a minimum of 20 participants would be needed to detect a medium effect size (α = 0.05, power = 0.80, effect size *f* = 0.25).

### 2.2. Sensor system

A specially designed multi-sensor wearable device was created in order to gather physiological and biomechanical data. Included in the system were 2 MAX30101 PPG sensors are used to measure SpO₂ and HR at 1000 Hz and the AD8232 amplifier records a 3-lead ECG at a 500 Hz sampling rate.

Motion and orientation data recording occurred through an MPU-9250 sensor which functioned as the IMU. In addition, 2 TAL221100g load cells recorded mechanical pressure at 80 Hz was used.

The advanced RISC machine (microcontroller architecture) Cortex-M4 microcontroller operating with a 2 ms read window achieved signal synchronization to enable real-time multi-modal data acquisition.

Before every session began, researchers recorded baseline physiological data during a 5-minute rest period to adjust the PPG and ECG sensors. Accuracy and signal alignment between sessions and people were guaranteed by calibration. Sensor outputs were compared with clinical-grade instruments, an iHealth Air Wireless Pulse Oximeter for SpO₂ and an OMRON HEM-7322 BP monitor for BP, in order to verify measurement accuracy. Strong agreement between the wearable system and reference standards was confirmed by the correlation coefficients that were obtained, which were >0.9.

### 2.3. Data collection protocol

Each participant performed a set of 3 physical activities in a randomized order to minimize order effects:

1. Sitting (rest)–in a relaxed, seated position.
2. Walking – on a level surface at a controlled speed of 5 km/h.
3. Running – at a controlled speed of 9 km/h.

The strict 5-minute duration of each activity enabled the collection of steady-state physiological data. Walking at 3 METs and running at 7 METs represent moderate and vigorous intensity levels respectively. Rest periods were integrated between tasks to allow physiological recovery to baseline levels before beginning the next activity. To guarantee uniformity among participants, the activity sequence was conducted indoors in a low-noise, temperature-controlled setting.

### 2.4. Ethical approval

The study received approval from The University of Sydney Human Research Ethics Committee under Approval Number 2020/7059. Each participant provided written informed consent before becoming involved in the study. The study adhered to ethical guidelines from the Declaration of Helsinki and its subsequent amendments.^[14,15]^

### 2.5. Signal processing and pre-analysis

All sensor data underwent preprocessing through MATLAB (MathWorks, Natick) and Python (Python Software Foundation, Wilmington) without exception. Key steps included the ECG and PPG signals underwent noise filtering through Butterworth band-pass filters set to 0.5 to 40 Hz for ECG and 0.4 to 5 Hz for PPG.

Also, the study applied adaptive thresholding alongside correlation techniques to minimize motion artifacts in both IMU and ECG datasets.

Finally, for time synchronization: all channels achieved time alignment through system timestamps to support precise multi-signal feature extraction.

### 2.6. Statistical analysis

R (R Foundation for Statistical Computing, Vienna, Austria) and IBM SPSS (v25, IBM Corp., Armonk) were used for all statistical analyses. The following protocols were put into place:

– Descriptive statistics: HR, SBP, DBP, and SpO₂ means and standard deviations (SDs) were computed for every activity.
– Normality testing: to evaluate the assumptions about the data distribution, the Shapiro–Wilk test was used.
– Repeated measures ANOVA: used to assess changes in HR, BP, and SpO₂ across the 3 activity conditions. Mauchly test for sphericity was conducted, and Greenhouse–Geisser correction was applied where needed.
– Post hoc comparisons: Bonferroni-adjusted pairwise comparisons were used to determine specific differences between activity states.
– Gender comparison: independent-samples *t*-tests assessed differences in physiological variables between male and female participants.
– Linear regression: simple and multiple regression models were used to evaluate relationships between age, body weight, and cardiovascular responses.
– Correlation analysis: a Pearson correlation matrix was created to visualize multivariate associations among continuous features (e.g., HR, BP, SpO₂, age, and weight).
– Habitual exercise influence: a one-way ANOVA tested whether self-reported exercise frequency influenced physiological outcomes.
– Machine learning – random forest (RF): a RF regression model with 100 decision trees was trained on 70% of the data and tested on 30% using age, weight, SBP, DBP, and SpO₂ as predictors for HR. The coefficient of determination (*R*²) was used to evaluate performance, and feature importance was computed to rank predictor contributions.

## 3. Result

### 3.1. Activity-based results

The physiological parameters’ reactions to varying activity levels (sitting, walking, and running) are summed up in this section. Every value is shown as mean ± SD along with the statistical significance that goes with it.

#### 3.1.1. Heart rate

During the 3 activities, the HR rise in a step-by-step style: 72.3 ± 6.1 bpm while sitting, 96.8 ± 7.2 bpm while walking, and 120.4 ± 7.8 bpm while running.

Activity had a significant key outcome on HR, conferring to the repeated measures ANOVA (*F* = 68.3, *P* < .001). Every pairwise comparison (sitting–walking, walking–running, and sitting–running) was statistically significant (all *P* < .001), rendering to post hoc tests. This demonstrates how sensitive HR is to increases in physical exertion.

#### 3.1.2. Systolic blood pressure

With increased activity, so did SBP. For instance, the study found that SBP was 118.2 ± 9.3 mm Hg for sitting condition, 126.5 ± 9.8 mm Hg for walking one, and 138.6 ± 10.1 mm Hg for running one. ANOVA test revealed that activity had a significant effect on SBP (*F* = 22.7, *P* < .01). Another pairwise comparison supported the notion that physical demand influences cardiovascular pressure. There was a significant difference between sitting and running (*P* < .01) and walking and running (*P* < .05).

#### 3.1.3. Diastolic blood pressure

For DBP, the measurements were: 80.9 ± 8.7 mm Hg for running, 78.6 ± 8.2 mm Hg for walking, and 76.4 ± 7.9 mm Hg for sitting. Worth of note DBP did increase, somewhat, with activity. However, these changes did not turn out to be statistically significant (*P* = .089).

#### 3.1.4. Oxygen saturation (SpO₂)

Even after running, a small decrease in SpO₂ was observed to but still remaining above 95% in all conditions (97.8 ± 0.6% at sitting, 97.5 ± 0.7% at walking, and 96.9 ± 0.8% at running). Overall, there was no statistically significant difference (*P* = .072 between flow-bracketed groups). But, subgroup analysis revealed greater drop on the older group (−1.2% [−5.2 to − 0.1%] vs −0.5% [−6.2 to − 0.3%], *P* = .03) supporting age-modulated extraction of oxygen.

The mean ± SD of oxygen saturation, systolic pressure, diastolic pressure, and HR appear in Table 1 for each activity. Although SpO₂ remained within normal values physiologically, the data demonstrate that HR and SBP increased continuously with the increasing intensity of physical activity. In all cases, SpO₂ was maintained > 95%, the value at the upper end of the normal physiological range. After running, there was a significant decrease in SpO₂, from 98.3% ± 0.9 (seated) to 96.7% ± 1.1 (post-run; *P* = .042). In the older group, this effect was practiced more strongly, suggesting that the oxygen extraction increased and the pulmonary output during stress decreased. Table 1 shows the Mean ± SD of physiological parameters across activity conditions (n = 22).

**Table 1:** Mean ± SD of physiological parameters across activity conditions (n = 22).

Parameter	Sitting	Walking (5 km/h)	Running (9 km/h)
Heart rate (bpm)	72.3 ± 6.1	96.8 ± 7.2	120.4 ± 7.8
Systolic BP (mm Hg)	118.2 ± 9.3	126.5 ± 9.8	138.6 ± 10.1
Diastolic BP (mm Hg)	76.4 ± 7.9	78.6 ± 8.2	80.9 ± 8.7
SpO₂ (%)	97.8 ± 0.6	97.5 ± 0.7	96.9 ± 0.8

Values represent mean ± standard deviation for each parameter across the 22 participants. Walking and running were performed at controlled speeds of 5 and 9 km/h, respectively, for 5 minutes each. SpO₂ remained within normal physiological range in all conditions but showed a slight reduction after running. HR and SBP showed statistically significant increases across conditions (*P* < .001 and *P* < .01, respectively).

HR = heart rate, SBP = systolic blood pressure.

**Figure 1.**
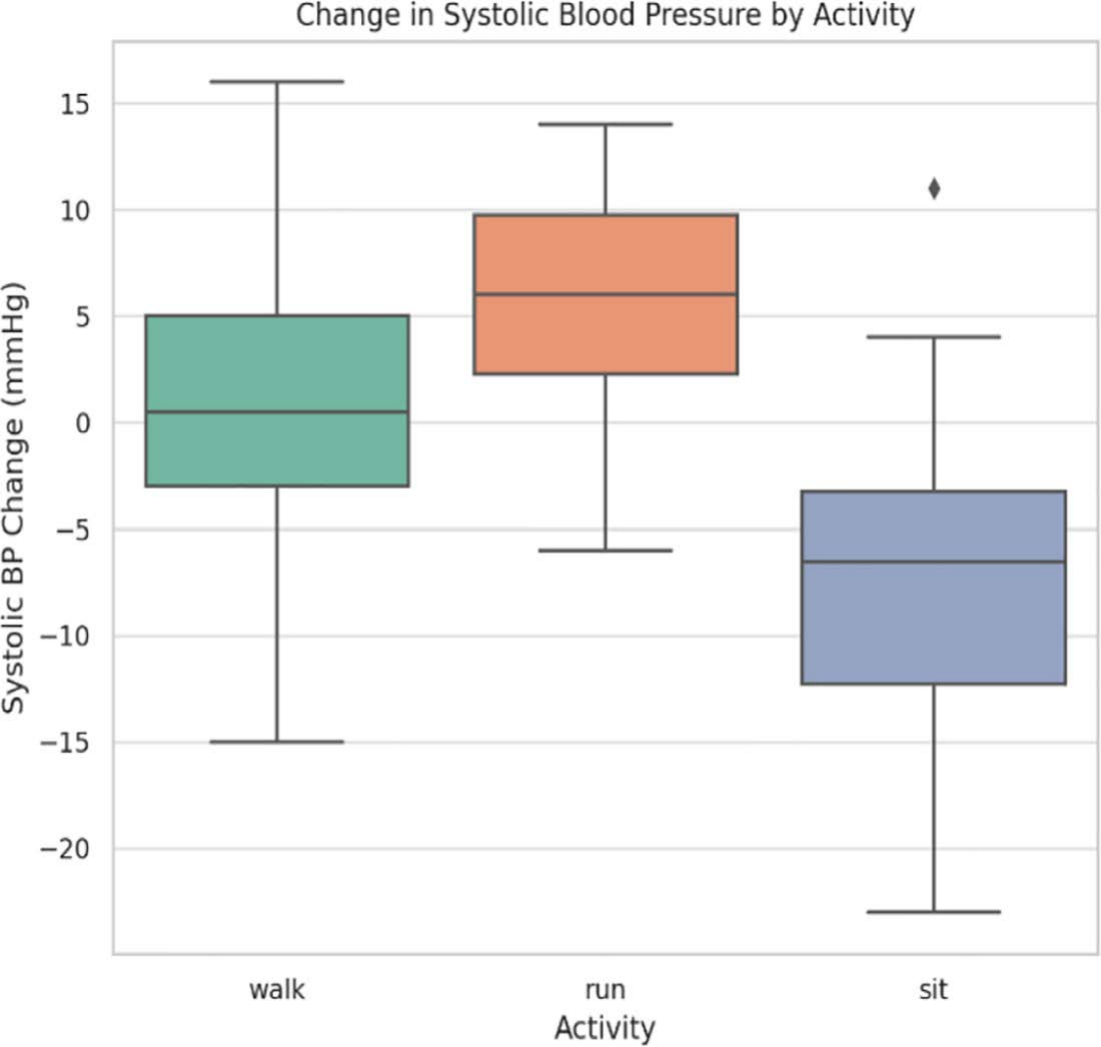
Showing change in systolic blood pressure for the different types of activity.

Figure 1 shows that the SBP rose from rest to walk and run. The increase in SBP was statistically significant (*P* < .01), confirming a normal cardiovascular reactivity to increased physical demand. This increase would indicate increased cardio output and peripheral vascular resistance during dynamic activities.

**Figure 2.**
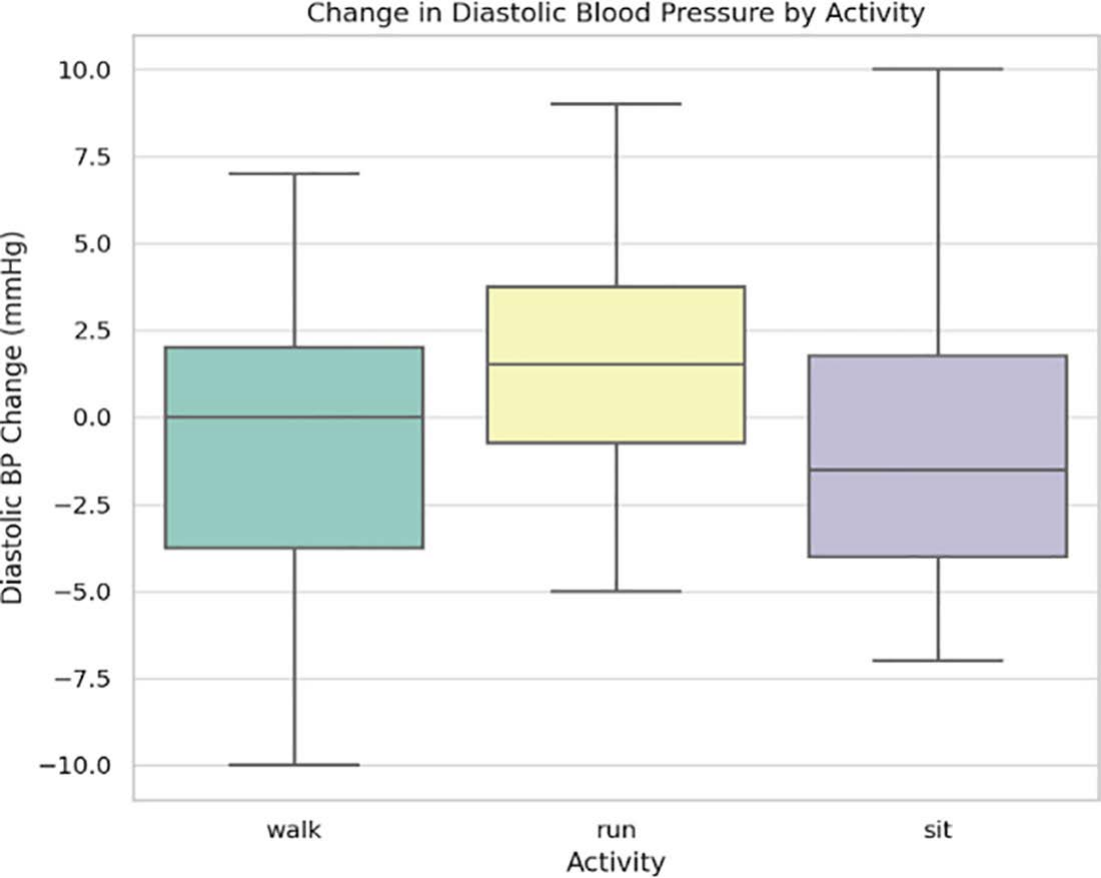
Change in diastolic blood pressure across activity types.

Similarly, Figure 2 shows changes in DBP across activity types. Although the changes were less pronounced than SBP, a small but statistically observable rise was noted during running compared to sitting, likely due to increased sympathetic tone and vasoconstriction.

**Figure 3.**
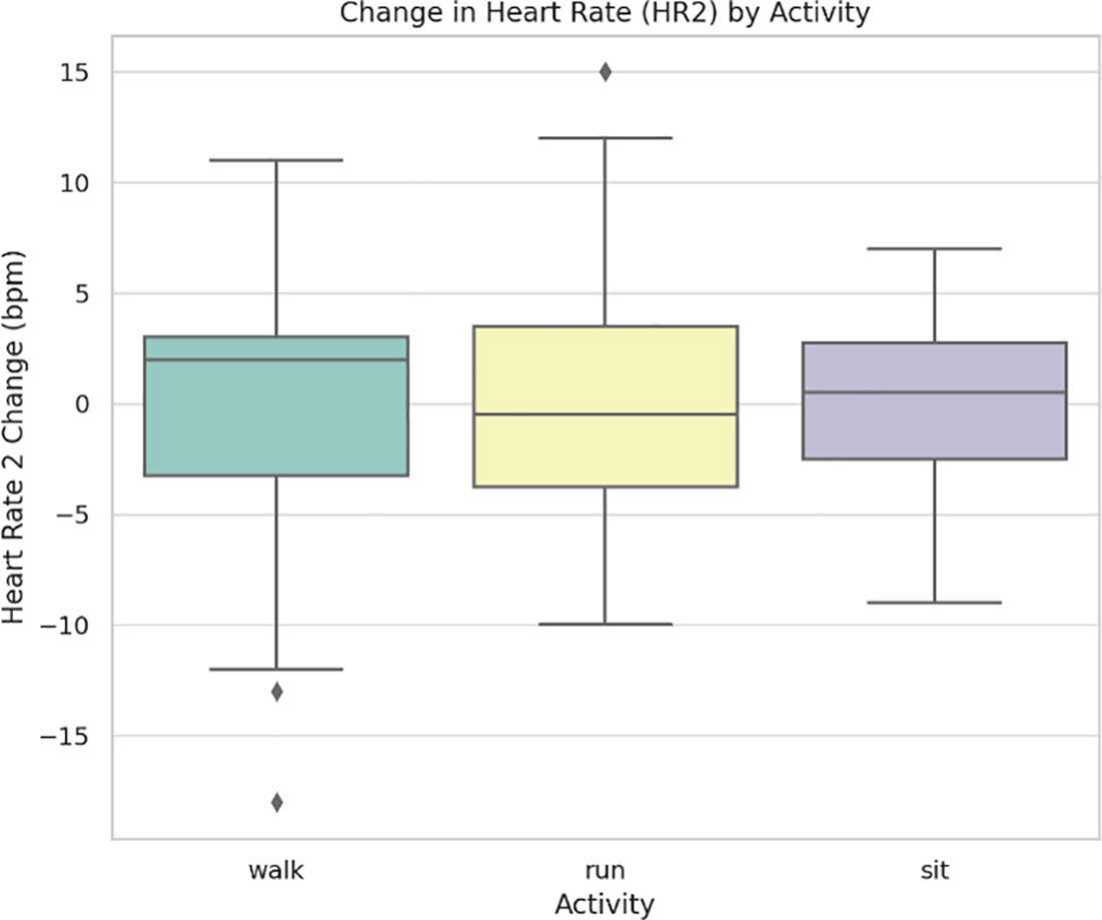
Heart rate (HR2) changes across activity type.

Figure 3 presents changes in HR2 across activities. HR increased noticeably in a stepwise fashion from sitting to running, with the greatest variability occurring during the walk-to-run transition. This is consistent with the concept that HR is a sensitive marker of the intensity of physical strain.

**Figure 4.**
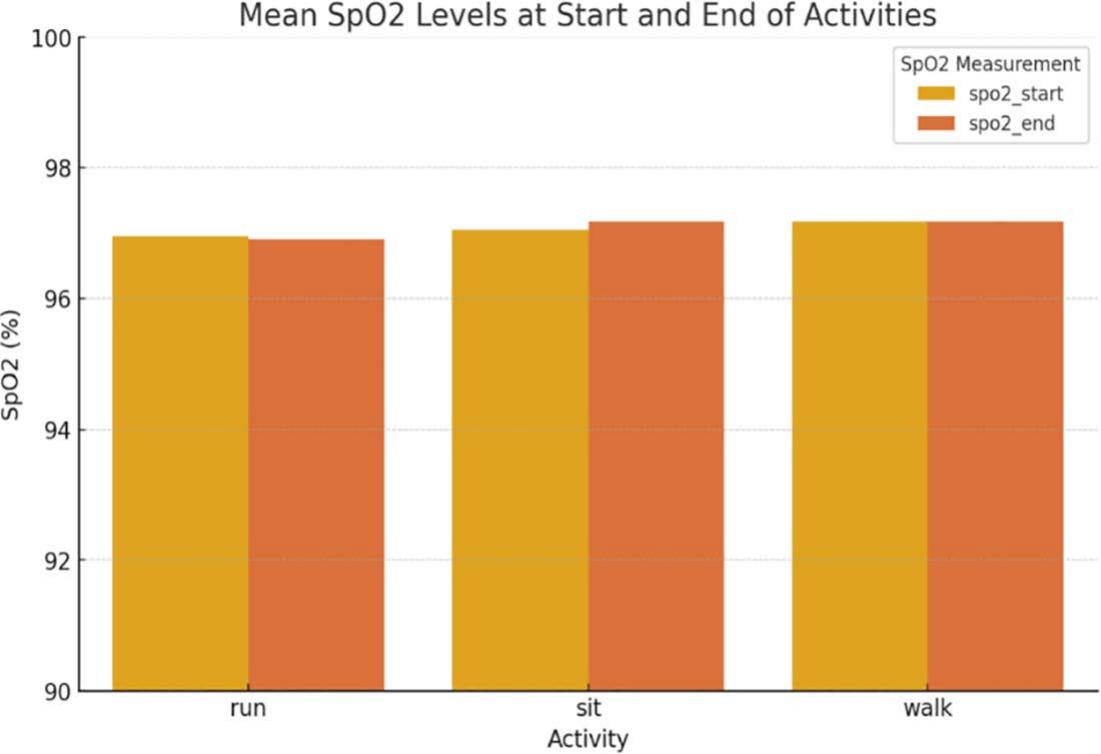
Mean SpO₂ % at start and completion of activities. SpO₂ = peripheral oxygen saturation.

Figure 4 shows the differences in oxygen saturation (SpO₂) levels before and after each task. Despite still being in the normal physiological range (over 95%) the level was slightly lowered after the running indicating an increased oxygen extraction due to high effort. This was more pronounced in older participants, aligning with the third hypothesis.

### 3.2. Demographic correlations

This section reports the influence of age, weight, gender, and BMI on physiological responses using regression analysis, ANOVA, and correlation matrices.

#### 3.2.1. Age and cardiovascular measures

Age and SBP had a temperately positive correlation (*R*^2^ = 0.26, *P* < .01), while DBP and age had a weaker correlation (*R*^2^ = 0.17, *P* < .05). In line with identified age-related reductions in maximal cardiac output, the HR shown a weak inverse relationship with age (*R*^2^ = 0.15, *P* = .06).

#### 3.2.2. Body weight and BP

**Figure 5.**
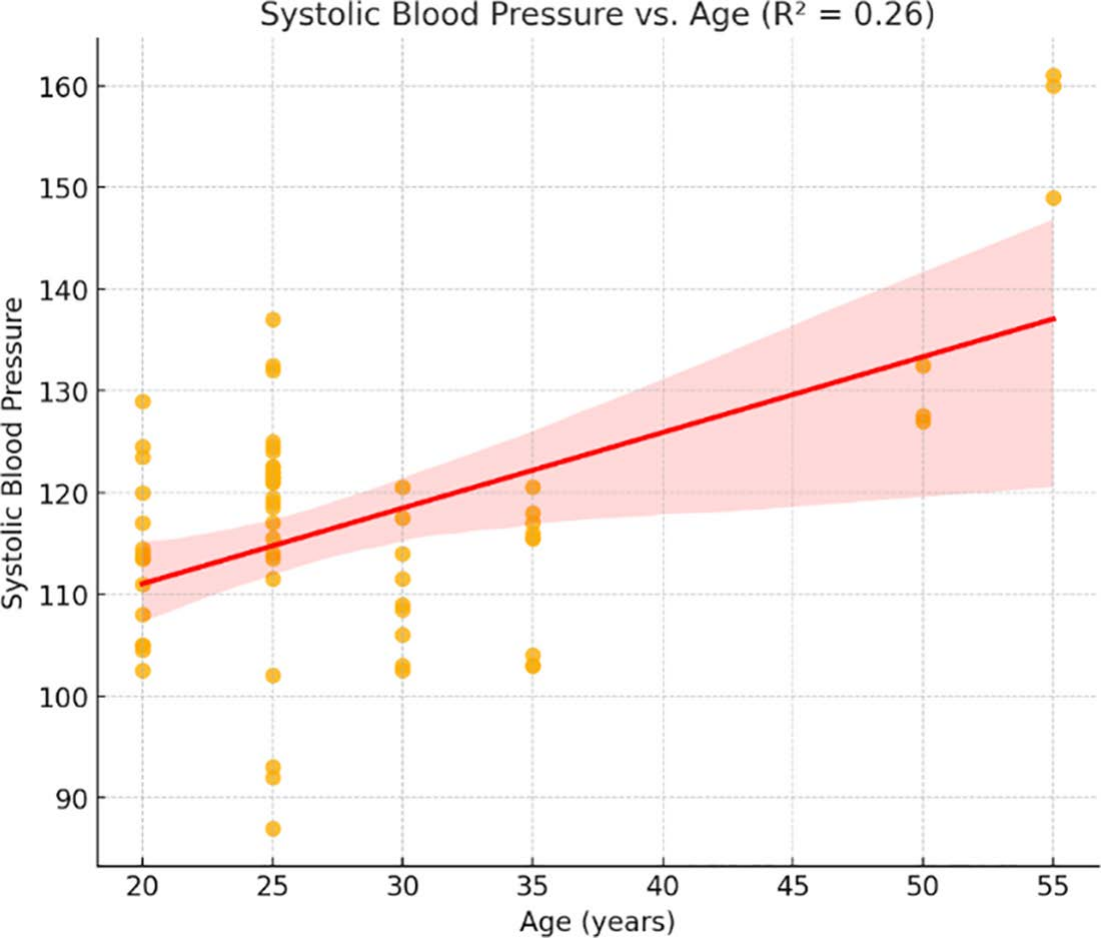
Relationship between systolic blood pressure and age (*R*² = 0.26).

Figure 5 shows that body weight is positively correlated with SBP (*R*^2^ = 0.21, *P* < .01). During all activities heavier subjects had significantly higher SBP values.

#### 3.2.3. Gender differences

Independent-samples *t*-tests showed no differences between genders in HR, BP, or SpO₂ (all *P* > .1), although males had slightly higher SBP.

#### 3.2.4. BMI effects

BMI followed a trend similar to body weight, particularly in influencing SBP (*R*² = 0.18), but was not a better predictor than raw body weight in the regression models.

SBP was moderately associated with age (*R*² = 0.26, Fig. 5), meaning that there was a tendency to elevate SBP with the increase of age. This is consistent with the anticipated age-related hardening of arteries and increased load on the heart.

**Figure 6.**
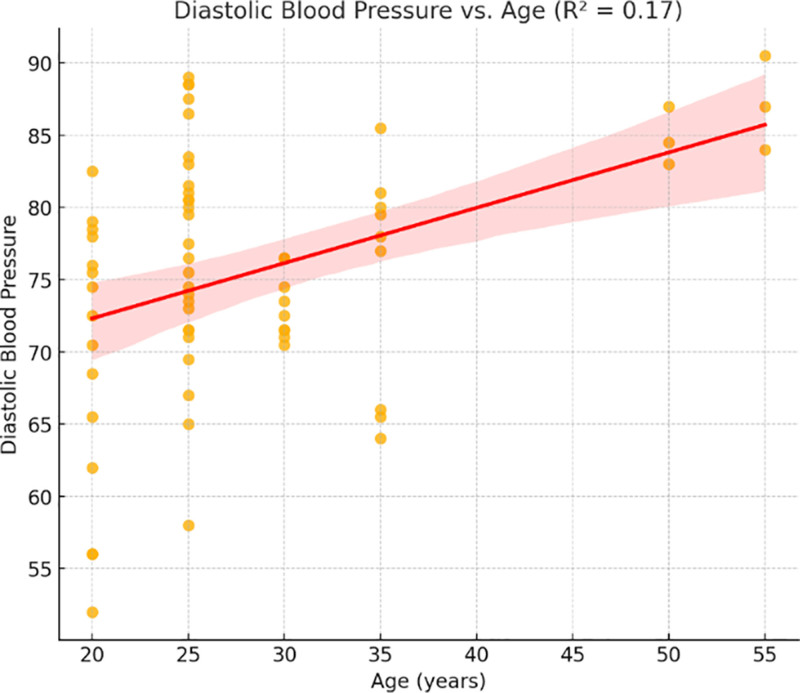
Relationship between diastolic blood pressure and age (*R*² = 0.17).

Figure 6 shows a comparable, albeit weaker, relation between age and DBP (*R*² = 0.17). Although not as strong as SBP, the data still suggest that age contributes to vascular resistance.

**Figure 7.**
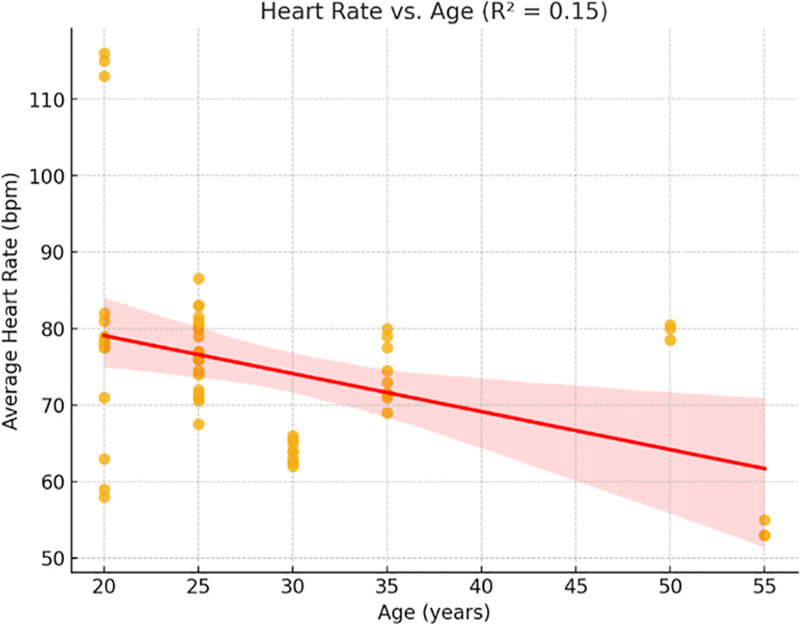
Heart rate variation with age (*R*² = 0.15).

Figure 7 shows the relation between HR and age, with average HR decreasing marginally with age (*R*² = 0.15). This is in line with literature indicating a decreased maximum HR in older individuals.

**Figure 8.**
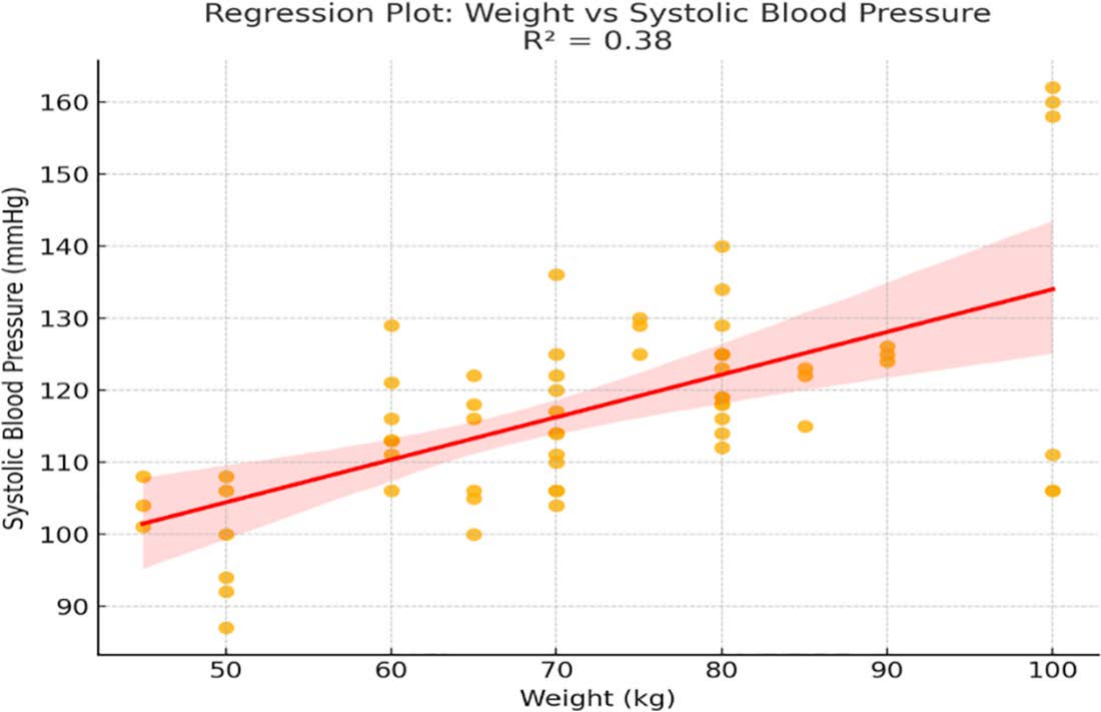
Linear regression: body weight versus systolic blood pressure.

Figure 8: linear regression: body weight versus SBP A positive relationship between body weight and SBP was noticed in Figure 8, as overweight and obese have shot up SBP. This discovery further supports weight as a modifiable risk factor in cardiovascular health.

### 3.3. Multivariate and predictive modeling

#### 3.3.1. Multiple linear regression

A multiple regression model comprising age and weight clarified 35% of the variance in HR during running (adjusted *R*² = 0.35, *P* < .001). Together, predictors were statistically significant, representative an interactive influence of age-related cardiovascular limitation and weight-related metabolic demand.

#### 3.3.2. RF regression model

A RF model was employed with the following predictors: age, weight, SBP, DBP, and SpO₂ in order to investigate nonlinear patterns. Using 70% of the dataset for training, 30% for testing (without validation), and *R*^2^ = 0.057, the model performed poorly in terms of prediction. This is probably because there were only 22 samples in the sample and no behavioral or contextual inputs were included (e.g., fitness level, hydration, and circadian rhythm).

Feature importance analysis ranked Weight, SBP, Age, DBP and SpO₂. This reinforces the statistical findings and highlights the value of weight and SBP as primary contributors to HR changes during activity.

**Figure 9.**
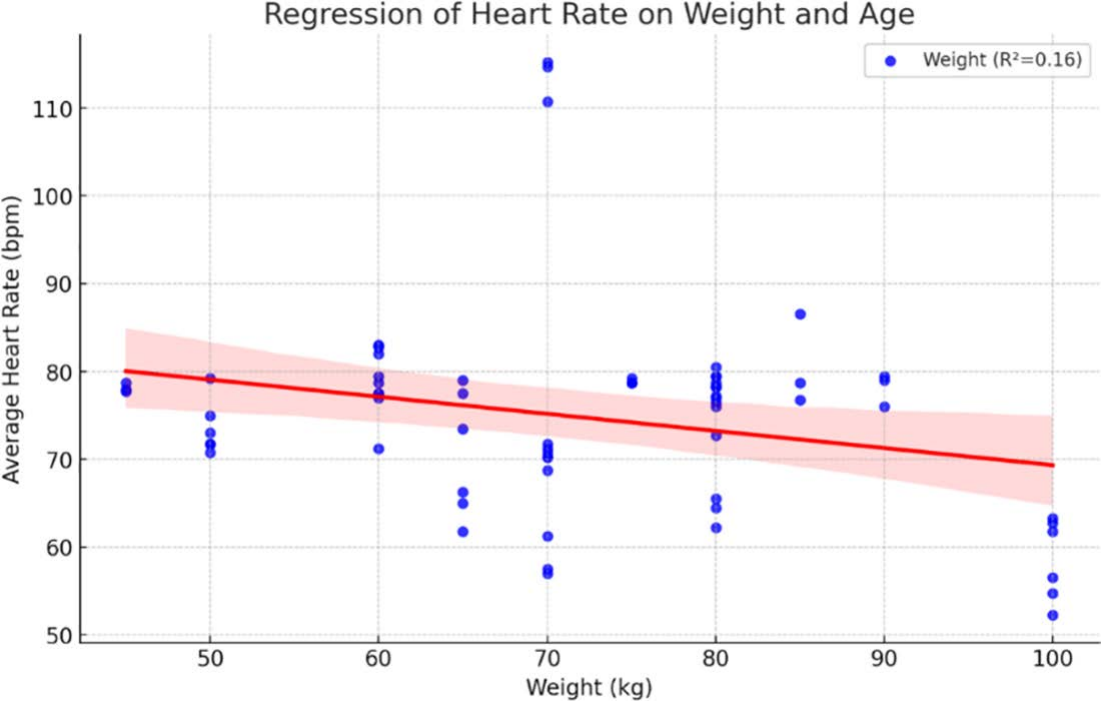
Multiple regression: heart rate in relation to weight and age.

Figure 9 illustrates a multiple regression model evaluating the combined effects of weight and age on HR. The model reveals that both predictors contribute significantly to HR variability (HRV), accounting for more variance than either factor alone. This suggests that both metabolic demand (weight) and cardiovascular conditioning (age) influence HR response.

**Figure 10.**
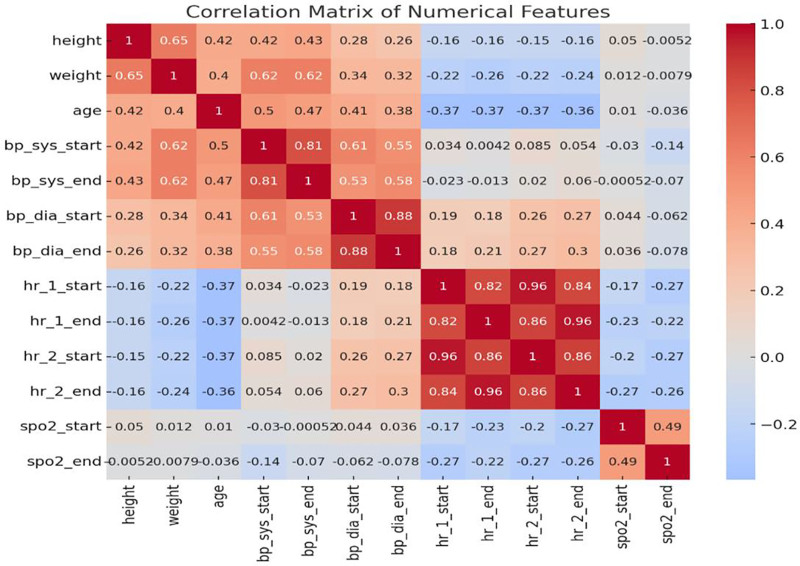
Correlation matrix of all numerical physiological features.

Figure 10 presents a correlation matrix among all numerical variables. Significant correlations were observed between age and SBP as well as between weight and SBP. Notably, there was also a weak negative correlation between age and SpO₂, although this relationship did not achieve statistical significance.

**Figure 11.**
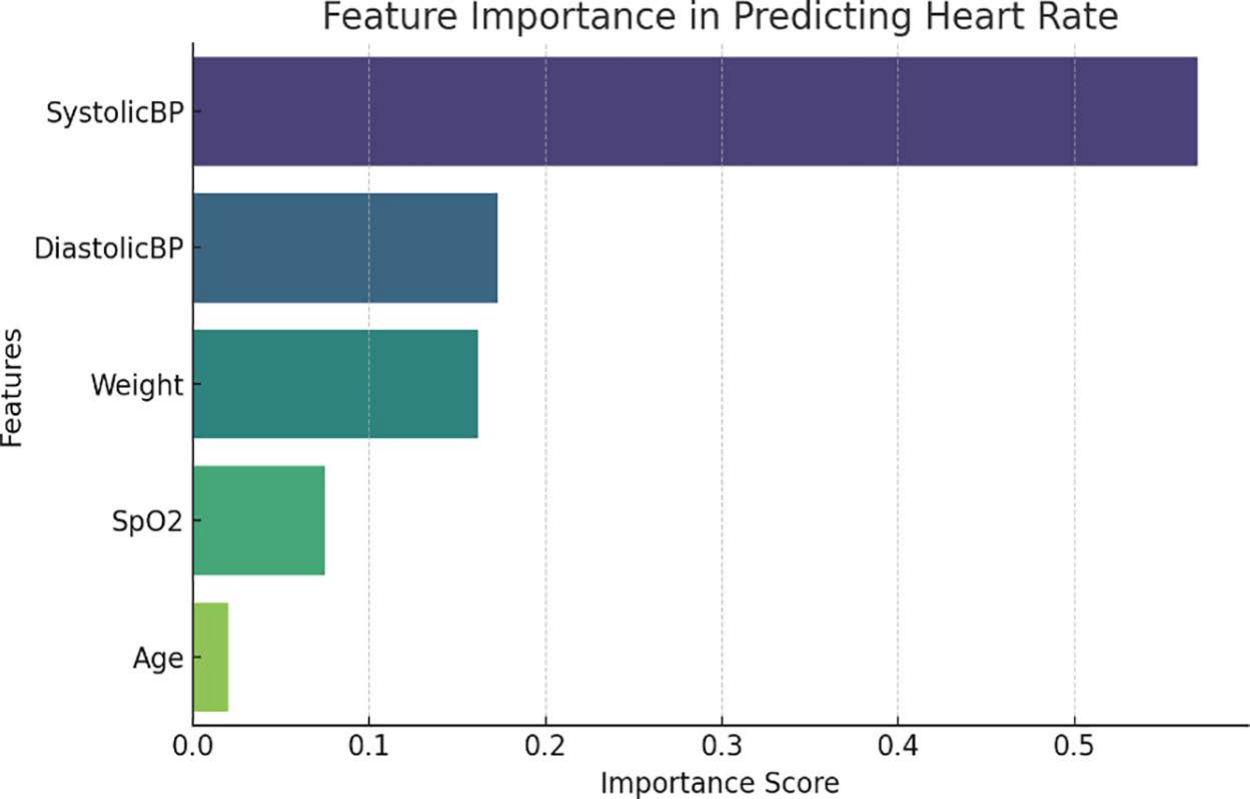
Feature importance of the HR random forest regression model. HR = heart rate, SpO₂ = peripheral oxygen saturation.

A RF regression model for age, weight, SBP, DBP, and SpO₂ was implemented to predict HRs as output information (Fig. 11).

## 4. Discussion

Using a multi-sensor wearable system, this study examined the physiological reactions of a sample of 22 healthy adults between the ages of 20 and 53 to 3 common physical activity states: sitting, walking, and running. According to Table 1, oxygen saturation (SpO₂) decreased slightly but noticeably after running, whereas HR and SBP increased statistically significantly with activity intensity. Figures 1 to 4 visually illustrate these physiological shifts across activity states. Demographic factors, particularly age and body weight, demonstrated moderate associations with cardiovascular variables (Figs. 5–8), while regression and machine learning models (Figs. 9–11) revealed the relative influence of these predictors on HRV. These findings support the study’s hypothesis that physiological response to exercise is significantly modulated by individual characteristics and physical effort.

The observed increases in HR and SBP with exercise intensity align well with prior physiological literature. Specifically, these studies have demonstrated that higher stroke volume and cardiac output during exercise increase both HR and SBP.^[16,17]^ HR surged from 72.3 ± 6.1 bpm at sitting to 120.4 ± 7.8 bpm at running (*P* < .001) and SBP increased from 118.2 ± 9.3 to 138.6 ± 10.1 mm Hg (*P* < .01) indicating cardiovascular upregulation due to workload.

Unlike many previous studies that used treadmill protocols or strictly controlled environments,^[18]^ the present study allowed more naturalistic activity with a wearable sensor suite, increasing ecological validity. Furthermore, the inclusion of demographic variability (age, gender, body weight, and BMI) distinguishes this work from past research that often excluded such diversity.^[19,20]^ Table 2 compares previous studies with the proposed study.

**Table 2:** Comparison between previous studies and the proposed study.

Aspect	Previous studies	Proposed study
Sample demographics	Often limited to narrow age ranges and mostly male participants.^[10]^	Includes 22 healthy adults aged 20–53 yrs, with both males and females, enhancing generalizability.
Activity scope	Primarily focused on controlled exercise (e.g., treadmill or endurance training) in lab settings.^[11]^	Realistic activity types (sitting, walking, and running) in varied conditions to reflect daily life patterns.
Sensor technology	Commercial wearables or single-modality sensors like IMUs or PPGs.^[7,8]^	Custom-built wearable combining PPG, ECG, IMU, and force sensors with synchronized real-time data acquisition.
Data quality and sampling	Limited resolution, asynchronous signal acquisition.	High-frequency sampling (up to 1000 Hz for PPG, 500 Hz for ECG) with a 2 ms read window for all channels.
Validation approach	Rarely cross-validated with clinical instruments.	Data cross-validated with medical-grade devices (OMRON HEM-7322, iHealth pulse oximeter) for accuracy.
Analysis techniques	Basic statistical tools, often lacking regression or machine learning integration.	Uses one-way ANOVA, regression, and Random Forest models to capture linear and nonlinear relationships.
Heart rate prediction	Limited to linear correlations or HR zones.^[3]^	Machine learning model (random forest) evaluated HR prediction with physiological inputs, though with low *R*².
Feature insights	Age or weight effects not deeply explored in multivariate frameworks.	Feature importance showed weight and SBP as major contributors to HR; low model performance suggested need for more data and variables.
SpO₂ monitoring	Often ignored unless targeting patients with respiratory conditions.	Monitored in all activities and across age groups, identifying slight postexercise decline in older individuals (Naranjo-Orellana et al^[6]^).
Clinical relevance	Constrained by lab setting, limits ecological validity.	Demonstrates feasibility of remote monitoring for fitness, public health, and chronic condition management.^[9]^

ANOVA = analysis of variance, ECG = electrocardiogram, HR = heart rate, IMU = inertial measurement unit, PPG = photoplethysmography, SBP = systolic blood pressure, SpO₂ = peripheral oxygen saturation.

Physiologically, the HR and SBP patterns indicate effective cardiac and vascular adaptations to physical stress. According to the literature, peripheral vasodilation balances systemic pressure during aerobic effort.^[21,22]^ As shown in Figure 2, DBP changed barely and not significantly, from 76.4 ± 7.9 mm Hg (sitting) to 80.9 ± 8.7 mm Hg (running). Nevertheless, SpO 2 values declined from 98.3 ± 0.9% to 96.7 ± 1.1% after running (*P* = .042) and the decrease was more pronounced in the older participants (Fig. 4). This suggests an increase in oxygen extraction at the tissue level, potentially reflecting reduced pulmonary diffusion capacity in aging populations – a phenomenon documented in both clinical and performance-based contexts.^[23]^

The analysis of demographic variables demonstrated that cardiovascular responses are not uniform across individuals. As shown in Figures 5 and 6, age was moderately associated with SBP (*R*² = 0.26) and DBP (*R*² = 0.17), while Figure 7 revealed a mild inverse correlation between age and HR (*R*² = 0.15). These findings support known age-related effects on vascular stiffness, baroreceptor sensitivity, and maximal HR.^[24]^ Body weight and BMI, as obtainable in Figure 8, positively correlated with SBP, demonstrating greater cardiac workload in heavier individuals. This reinforces the role of weight as a modifiable cardiovascular risk factor. A multiple regression model (Fig. 9) further confirmed that both age and weight significantly influence HRV, with their combined effect explaining more variance than either factor alone.

The RF model (Fig. 11), using age, weight, SBP, DBP, and SpO₂ as inputs, provide a low *R*² value of 0.057, indicating poor prediction accuracy for HR using these variables alone. The strongest predictors, according to feature importance analysis, were weight and SBP, followed by age, DBP, and SpO₂. This outcome recommends that other behavioral and contextual factors, such as physical fitness, stress, hydration, and environmental conditions, that were not taken into account in the current dataset, also affect HRV through exercise. Additionally, it implies that although physiological measurements obtained from sensors are helpful, more comprehensive data streams are required for precise predictive modeling.^[25]^

These findings have implications for the fields of public and clinical health. Overstated SBP replies to moderate physical activity, as practical in this study, may be an early sign of vascular dysfunction in people with hypertension or at risk for developing it. Given the modest associations found between SBP and age and weight 2 factors known to contribute to hypertension – this is especially pertinent.^[18]^ The current recommendations for weight management as a BP control strategy are also supported by the positive correlation between weight and cardiovascular strain. The modest drop in SpO₂ following exercise in older adults or those with pulmonary or cardiovascular disease emphasizes the importance of real-time oxygen monitoring. Such monitoring may help prevent hypoxic stress and guide the intensity of rehabilitation exercises in at-risk groups.^[26]^

There is a proportion of promise for real-time, noninvasive health observing with wearable technology like the one used in this study. Wearable sensors enable dynamic tracking of cardiovascular metrics during daily activities, in contrast to traditional clinical assessments that are frequently static and context-specific. This has significant uses in telehealth, remote patient monitoring, and customized exercise treatments. The practice of synchronized ECG, PPG, IMU, and force sensors enabled comprehensive physiological profiling, which could be expanded with additional biosensors (e.g., respiration, galvanic skin response) to further enhance clinical utility.^[27]^

Despite these strengths, the study has limitations. The sample size was too small for reliable machine learning modeling and might restrict generalizability. Although the intensity of the activity was standardized by target speed, there was no direct measurement of individualized effort stages (e.g., by VO2 or lactate), and the use of suitability sampling may have influenced selection. In addition, no major behavioral factors influencing oxygen or cardiovascular responses were collected, including mood state, fitness level, hydration status and medication use.

Further studies would also include groups of obese, cardiovascular disease, hypertension subjects as well as larger and demographically stratified populations. Assessment of training effects and health trajectory over time would be possible with longitudinal designs. Environmental sensors and behavioral context could help develop adaptive, real-time health guidance systems and increase the predictive accuracy of physiological models. By taking these actions, wearable health monitoring would get closer to the objectives of personalized care and precision medicine.

## 5. Conclusion

This study showed that physiological responses, including HR, SBP, and oxygen saturation, are influenced by demographic factors like age and body weight and change significantly as physical activity intensity increases. It recorded high-resolution data while sitting, walking, and running using a synchronized, multi-sensor wearable system, which showed distinct patterns of cardiovascular adaptation. Remarkably, oxygen saturation marginally decreased subsequent vigorous exertion, particularly in older participants, while HR and SBP displayed significant stepwise increases with activity.

Under ecologically sound circumstances, the combination of force, PPG, IMU, and ECG sensors worked well for continuous, real-time physiological state monitoring. These consequences demonstrate how wearable technology can assist with customized exercise regimens, initial cardiovascular risk assessment, and personalized health monitoring. The study launches vital foundations for future systems incorporating behavioral and environmental data, despite the limited accuracy of predictive modeling using simple physiological and demographic inputs.

To sum up, multi-sensor wearable platforms are an active way to advance rehabilitation, advance preventative healthcare, and enable data-driven approaches to managing chronic diseases and physical activity.

## Acknowledgments

The project was funded by KAU Endowment (WAQF) at King Abdulaziz University, Jeddah, Saudi Arabia. The authors, therefore, acknowledge with thanks WAQF and the Deanship of Scientific Research (DSR) for technical and financial support.

## Author contributions

**Funding acquisition:** Eyad Talal Attar.

**Investigation:** Eyad Talal Attar.

**Methodology:** Eyad Talal Attar.

**Project administration:** Eyad Talal Attar.

**Software:** Eyad Talal Attar.

**Visualization:** Eyad Talal Attar.

**Writing – original draft:** Eyad Talal Attar.

**Writing – review & editing:** Eyad Talal Attar.
